# Inflammatory mass or malignancy? The radiology of fish bone phlegmon: a review of 3 cases

**DOI:** 10.1093/bjrcr/uaag030

**Published:** 2026-07-28

**Authors:** Michel Alhilani, Larne Jones-Whiting, Sharmin Malekout, Abdelhamid Elshawarby, Andrew J Hayes, Eleanor Moskovic

**Affiliations:** Department of Radiology, The Royal Marsden NHS Foundation Trust, London, SW3 6JJ, United Kingdom; Department of Radiology, St. George’s University Hospitals NHS Foundation Trust, London, SW17 0QT, United Kingdom; The Sarcoma, Melanoma and Rare Tumours Unit, Department of General Surgery, The Royal Marsden NHS Foundation Trust, London, SW3 6JJ, United Kingdom; The Institute of Cancer Research, London, SM2 5GP, United Kingdom; Department of Radiology, The Royal Marsden NHS Foundation Trust, London, SW3 6JJ, United Kingdom; The Sarcoma, Melanoma and Rare Tumours Unit, Department of General Surgery, The Royal Marsden NHS Foundation Trust, London, SW3 6JJ, United Kingdom; The Sarcoma, Melanoma and Rare Tumours Unit, Department of General Surgery, The Royal Marsden NHS Foundation Trust, London, SW3 6JJ, United Kingdom; The Institute of Cancer Research, London, SM2 5GP, United Kingdom; Department of Radiology, The Royal Marsden NHS Foundation Trust, London, SW3 6JJ, United Kingdom

**Keywords:** soft tissue neoplasms, sarcoma, foreign bodies, fish bones, abdominal wall, phlegmon, diagnostic imaging, CT, MRI

## Abstract

Soft-tissue sarcomas of the abdomen and abdominal wall are rare, and their imaging appearances can overlap with those of benign inflammatory conditions. Foreign body-related phlegmon caused by a migrated fish bone from the bowel represents an uncommon but important diagnostic mimic, often indistinguishable from malignancy on cross-sectional imaging. Although several single case reports describe this entity, no radiology-focused multi-case series has been previously published. We present 3 patients referred to a regional sarcoma service with abdominal wall masses initially suspected to represent soft-tissue sarcoma. All 3 underwent multimodality imaging, which demonstrated heterogeneous, enhancing abdominal-wall lesions with associated inflammatory change. In each case, thin-slice CT ultimately identified a subtle linear hyperdense structure, corresponding to a migrated fish bone traversing or lying adjacent to bowel, establishing the correct diagnosis of foreign-body-induced phlegmon. MRI appearances were non-specific and mimicked neoplastic processes. Management varied from conservative antibiotic therapy to incision and drainage, with all patients making a full recovery and no recurrence. This case series highlights a rare but significant radiologic pitfall in the assessment of abdominal wall masses. Key learning points include scrutinizing CT in thin slices and bone windows for linear calcified foreign bodies, recognizing that MRI and PET-CT may mislead toward malignancy, and maintaining foreign-body phlegmon within the differential diagnosis for atypical abdominal wall “tumours.” Awareness of this entity can prevent unnecessary biopsies, oncologic referrals, and radical surgeries.

## Introduction

Soft-tissue sarcomas are rare malignant neoplasms of mesenchymal origin, accounting for less than 1% of adult cancers, and are recognized for their imaging heterogeneity and broad differential diagnosis.^1^ Radiological assessment is challenging, as benign inflammatory and infective conditions can closely mimic sarcoma on cross-sectional imaging.^2^ Despite advances in histopathological and molecular classification, imaging characteristics such as morphology and enhancement patterns frequently overlap between malignant and non-neoplastic processes, contributing to diagnostic uncertainty.^3^

Inflammatory and infectious processes of the anterior abdominal wall represent an important group of sarcoma mimics. Among these, foreign body-related phlegmon caused by transmural bowel perforation from an ingested fish bone is an uncommon but deceptive entity. Although most ingested fish bones pass uneventfully, a sharp fragment may perforate the gastrointestinal tract and migrate extraluminally. Subsequent propagation along fascial planes can result in a focal abscess or phlegmon within the abdominal wall, presenting as a mass-like lesion on imaging. While similar migratory processes have been described in other locations such as the pancreas or urachal region, abdominal wall involvement is particularly relevant to the differential diagnosis of soft-tissue tumors.^4–7^ These inflammatory lesions may demonstrate avid enhancement on MRI and increased uptake on fluorodeoxyglucose positron emission tomography-computed tomography (FDG PET-CT), producing an imaging appearance that closely resembles malignancy, especially when the patient does not recall foreign body ingestion.^5–7^

This diagnostic pitfall reflects the broader challenge of differentiating infection from malignancy. In a large tertiary referral series, radiological assessment failed to reliably distinguish infection from tumor in 59.7% of osteomyelitis cases and 81.5% of soft-tissue infections.^8^ Recognition of a subtle linear hyperdense structure representing the fish bone on thin-slice CT is therefore crucial, as it provides a key diagnostic clue and may prevent unnecessary oncologic referral, biopsy, or radical surgery.^6^^,^^9^^,^^10^

We present 3 cases of anterior abdominal wall phlegmon secondary to migrated fish bones, each initially suspected to represent a soft-tissue sarcoma. This radiology-focused case series highlights the imaging features that aid accurate diagnosis and underscores the importance of including foreign body related inflammation in the differential diagnosis of atypical abdominal wall masses.

## Case presentation

Written informed consent was obtained from all patients for publication of their clinical details and imaging findings. All images and data were anonymized in accordance with institutional and journal requirements.

### Case 1

A 71-year-old male patient presented to his GP with a 7-day history of a progressively enlarging, tender anterior abdominal-wall swelling associated with erythema and warmth. He reported frequent fish consumption but denied recent trauma or recollection of foreign body ingestion. A 7-day course of oral antibiotics prescribed by his general practitioner failed to improve symptoms, prompting hospital referral.

On examination, there was a firm, fluctuant periumbilical mass with overlying erythema and localized tenderness, but no systemic features of sepsis. Laboratory investigations demonstrated markedly elevated inflammatory markers (C-reactive protein 203 mg/L; erythrocyte sedimentation rate 110 mm/hr). Liver enzymes showed mild, fluctuating transaminitis (ALT 75 U/L; ALP 157 U/L), with otherwise preserved hepatic function. Remaining blood parameters were within normal limits.

**Figure 1:**
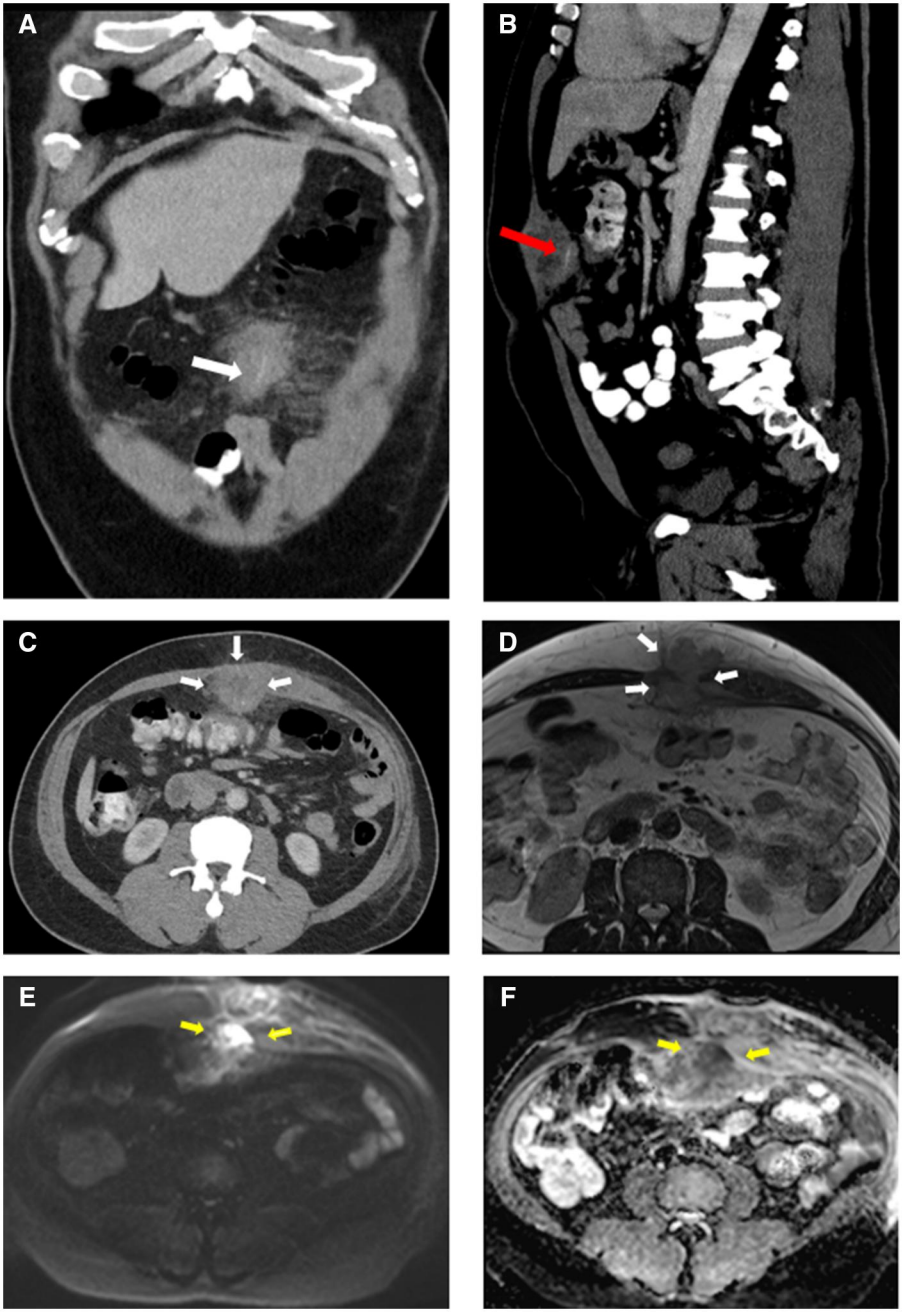
Case 1. Coronal (A), sagittal (B), and axial (C) CT with PV contrast as well as axial T1 MRI images (D) showing the abdominal wall phlegmon (white arrows) and a linear hyperdense fish bone (red arrow) penetrating the transverse colon and extending into the left rectus sheath. DWI B800 (E) and ADC map (F) show areas of marked diffusion restriction within the inflammatory mass.

Initial contrast-enhanced CT of the abdomen and pelvis revealed a heterogeneous, mass-like lesion within the anterior abdominal wall, tethering to the transverse colon, extending into the left rectus sheath, with surrounding inflammatory change. The lesion measured 56 × 38 mm and contained a central hypodense component of 21 × 22 mm. These features raised concern for a soft-tissue sarcoma, and the case was referred to the regional sarcoma multidisciplinary team (MDT) for specialist review (Figure 1).

Expert radiological reassessment identified a subtle linear hyperdense focus within the lesion, consistent with a migrated fish bone, appearing to traverse the transverse colon and extend into the anterior abdominal wall. Thin-slice reformats demonstrated a slender, approximately 2.3-cm calcified fragment (Figure 1). In light of this finding, the appearances were reinterpreted as an inflammatory phlegmon secondary to fish bone perforation with early enterocutaneous fistulation. The patient was initially managed conservatively with antibiotics and close observation.

Three weeks later, he re-presented with recurrent abdominal wall pain, pyrexia, and persistently elevated inflammatory markers. Examination revealed spontaneous fistulation of the lesion with purulent discharge through the overlying skin. He subsequently underwent incision and drainage of the abscess in theater, during which a fish bone was confirmed intraoperatively. Purulent material was sent for microbiological analysis, which demonstrated heavily bloodstained pus with abundant white cells (+++) and Gram-positive cocci.

**Figure 2:**
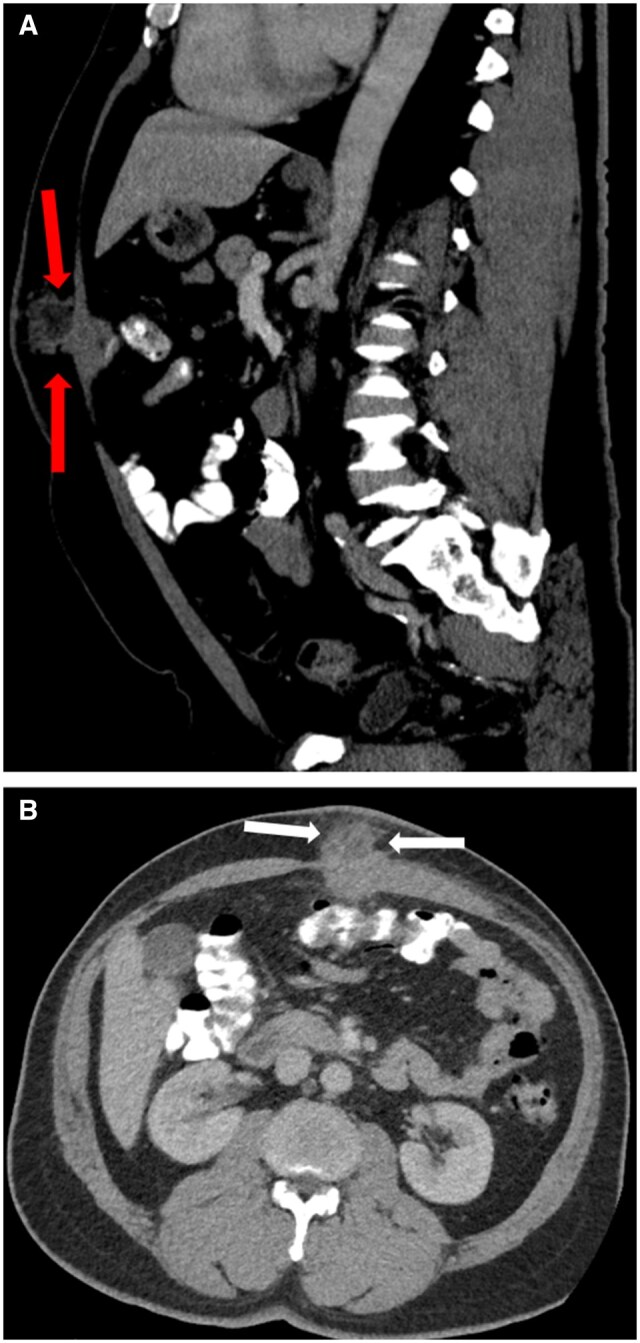
Case 1. Follow-up CT with PV contrast in sagittal (A) and axial (B) views demonstrates post-incision and drainage appearance of the inflammatory mass, which appears less bulky and avidly enhancing compared to Figure 1.

Post-procedural recovery was uneventful, with rapid symptomatic improvement. Interval CT demonstrated near-complete resolution of the inflammatory mass and substantial reduction in rectus-sheath edema (Figure 2). At subsequent clinical follow-up after 15 months, the patient remained well, with no evidence of recurrence or residual fistula formation.

### Case 2

A 42-year-old female patient presented to A&E with a rapidly enlarging anterior abdominal wall swelling that had developed over 7-14 days. She reported localized discomfort and mild tenderness but denied trauma, prior abdominal surgery, or recollection of foreign body ingestion. There were no systemic symptoms, including fever, night sweats, or weight loss. On examination, a firm, mildly tender anterior abdominal wall mass was palpable without overlying erythema or fluctuance.

Laboratory investigations demonstrated a modest inflammatory response: C-reactive protein 86 mg/L, white cell count 10.8 × 10^9^/L with mild neutrophilia (7.9 × 10^9^/L), and erythrocyte sedimentation rate 62 mm/hr. Liver enzymes showed mild transaminitis (ALT 54 U/L; ALP 128 U/L) with normal bilirubin, renal function, and remaining hematological indices. Overall, the biochemical profile was consistent with localized inflammation without systemic sepsis or end-organ dysfunction.

**Figure 3:**
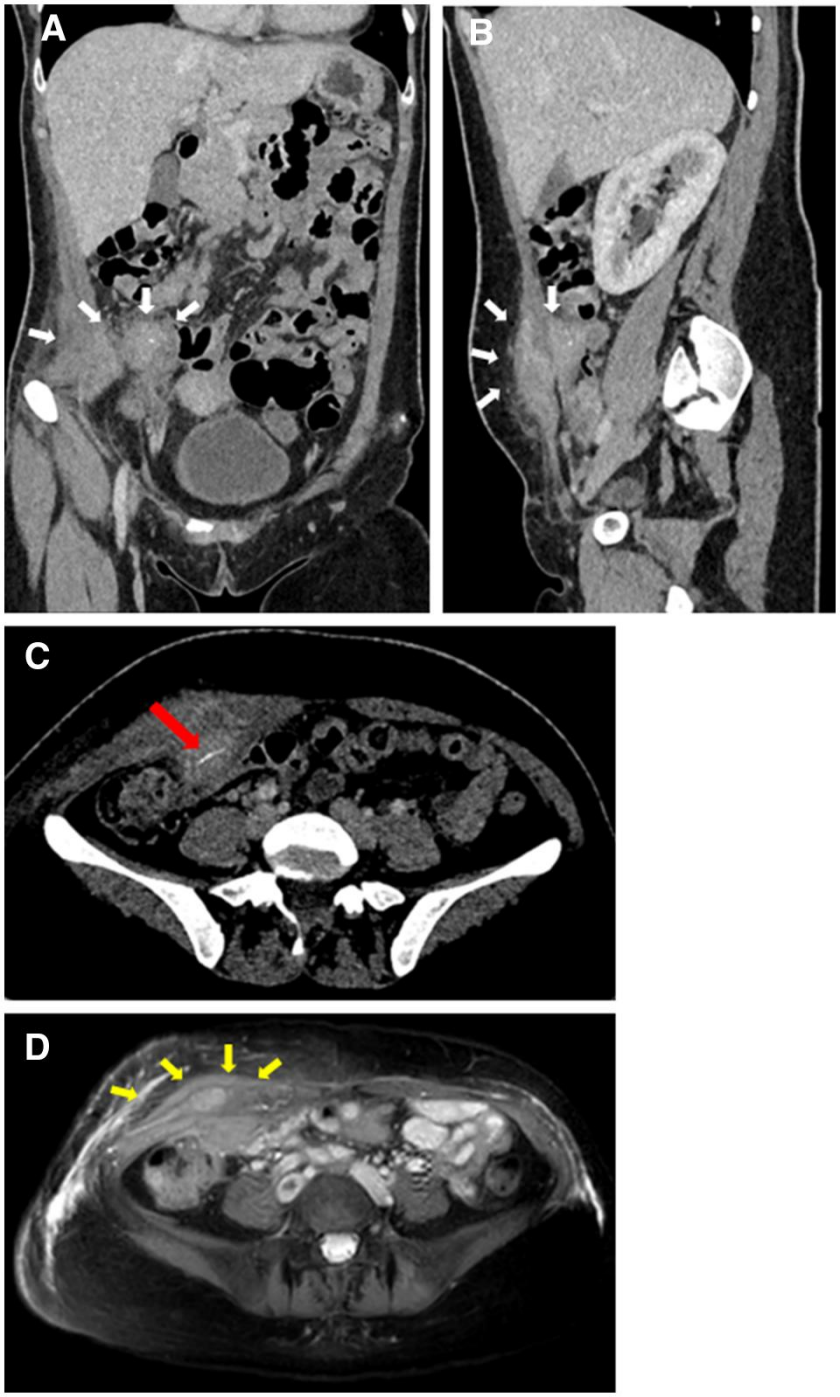
Case 2. Coronal (A), sagittal (B) and axial (C) CT with PV contrast as well as axial MRI T2 fat saturated (D) images showing the abdominal wall phlegmon (white arrows) and a linear hyperdense fish bone within (red arrow). The phlegmon displays a high signal on MRI T2FS images indicating edema with pockets of fluid along the right anterior subcutaneous tissues.

Contrast-enhanced CT of the abdomen and pelvis demonstrated a 45 × 25 mm inflammatory collection containing a linear hyperdense structure compatible with a retained fish bone, with associated fat stranding but no clear bowel communication or metastatic disease (Figure 3). Given the indeterminate nature of the lesion and the presence of incidental hepatic lesions, an MRI of the liver and pelvis was subsequently performed for further characterization. MRI demonstrated a well-defined lesion within the rectus sheath with heterogeneous T2 hyperintensity and peripheral enhancement following gadolinium administration (Figure 3). The appearances raised concern for a soft-tissue sarcoma or desmoid-type fibromatosis. The hepatic lesions were proven to be hemangiomata.

The case was reviewed at the regional Sarcoma MDT, where consensus interpretation favored a fish bone-related abscess or phlegmon masquerading as a neoplastic process. Conservative management with antibiotic therapy and close clinical monitoring was recommended.

The patient received intravenous, then oral antibiotics over 10 days, with rapid symptomatic improvement. No drainage or biopsy was required. At the 2-year review, the patient remains well with no recurrence.

### Case 3

A 52-year-old female patient presented to A&E with a 2- to 3-week history of right-sided abdominal pain and swelling. Her medical history included type 2 diabetes mellitus, beta-thalassemia trait, hypertension, hypercholesterolemia, and multiple prior abdominal operations (2 cesarean sections and 2 myomectomies). She had a BMI of 29 kg/m^2^, was a nonsmoker, and reported a high fish intake, stating she “loves fish.” She denied any foreign body ingestion, umbilical procedures, or recent trauma.

On initial review, she described intermittent periumbilical pain exacerbated by bending, associated with localized tenderness and swelling, but no systemic symptoms. Laboratory testing demonstrated a raised C-reactive protein of 53 mg/L, with all other parameters unremarkable.

**Figure 4:**
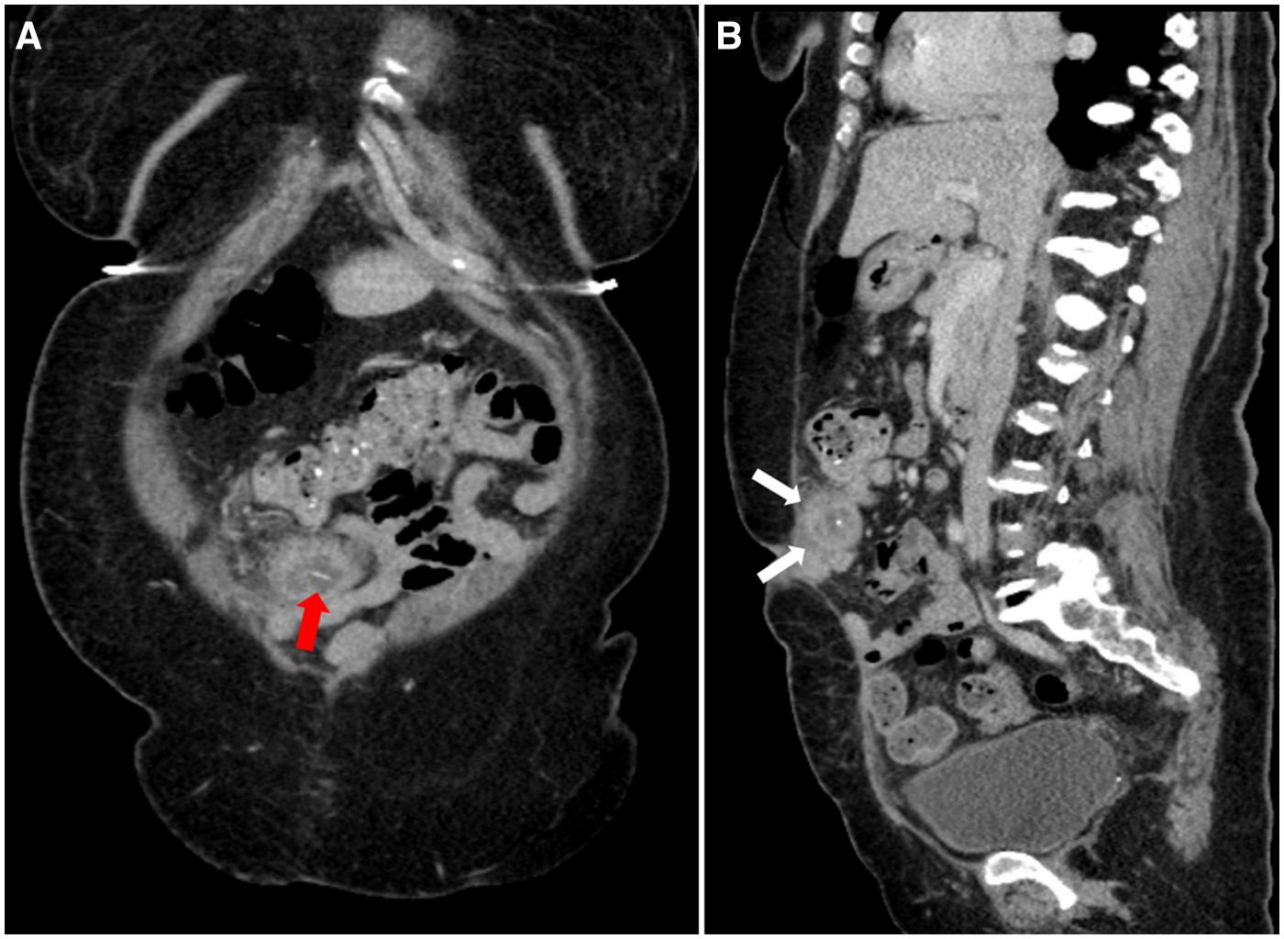
Case 3. Coronal (A) and sagittal (B) CT with PV contrast showing the retro-umbilical inflammatory mass (white arrows) and a linear hyperdense fish bone within the mass (red arrow).

Contrast-enhanced CT of the abdomen and pelvis demonstrated a 63-mm retro-umbilical lesion immediately superior to the umbilicus (Figure 4). The mass exhibited a thick, peripherally enhancing wall with a central low-attenuation component containing a linear hyperdense structure, highly suggestive of a foreign body. Surrounding inflammatory fat stranding was present, though there was no clear communication with adjacent bowel loops. The differential diagnosis at this stage included an inflammatory mass, urachal remnant, or soft-tissue sarcoma. An interval MRI was also arranged at time of presentation to further characterize the lesion and exclude an underlying neoplastic process, particularly desmoid-type fibromatosis given her history of prior abdominal surgeries.

**Figure 5:**
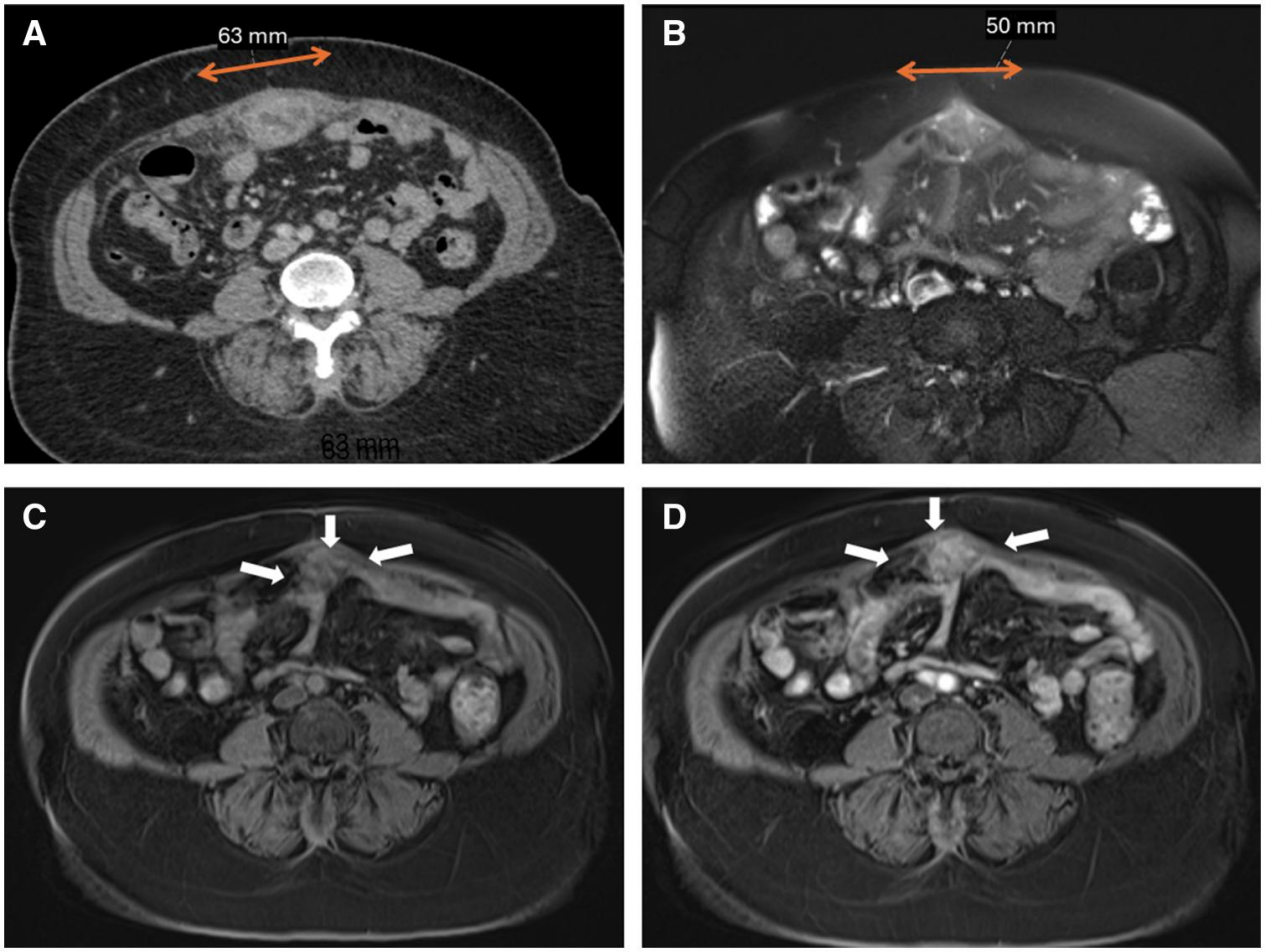
Case 3. Axial (A) CT with PV contrast image showing 63-mm anterior abdominal wall inflammatory mass, followed by an axial T2 fat saturated image (B) from the MRI acquisition performed 1 month following the CT, showing reduction in size. The mass was still displaying enhancing components on MRI, as seen on T1 fat saturated pre-gad (C) and post-gad (D) imaging.

She was commenced empirically on antibiotics, with rapid improvement in symptoms. The MRI performed 1 month later (Figure 5) demonstrated an interval reduction in lesion size to 50 mm. The lesion displayed intermediate T2 hyperintensity, low T1 signal, and heterogeneous post-contrast enhancement, with mixed diffusion restriction. The adjacent bowel appeared normal. Given her extensive surgical history, desmoid-type fibromatosis was considered, although a retained suture or foreign body remained possible.

The case was subsequently reviewed at the regional Sarcoma MDT. Consensus interpretation described a retro-umbilical phlegmon measuring 63 × 31 mm in April and 50 × 25 mm in June, caused by perforation of adjacent bowel loops by a centrally located fish bone. Both specialist radiologists and sarcoma surgeons agreed that the appearances were inflammatory rather than neoplastic.

The patient’s symptoms resolved completely without surgical intervention. At 1-year telephone follow-up, she remained well and reported no palpable or visible umbilical mass.

## Discussion

### Pathogenesis and previous case reports

Fish bone-related inflammatory processes represent a well-recognized but frequently overlooked cause of tumor-like lesions on cross-sectional imaging.^4–18^ Several authors describe a common pathophysiological mechanism underlying these presentations, which explains their ability to mimic malignancy. Following ingestion, a sharp fish bone may become impacted within the gastrointestinal tract and gradually erode through the bowel wall, often without causing acute peritonitis.^4^^,^^10^ The perforation site subsequently seals due to local inflammation, omental coverage, or fibrin deposition, allowing the foreign body to migrate extraluminally into adjacent soft tissues.^9^^,^^10^^,^^16^ Progressive inflammatory reaction around the migrated fragment can then lead to the formation of a focal abscess or phlegmon, which may extend along fascial planes toward the abdominal wall and present as a mass-like lesion on imaging.^4^^,^^9^^,^^17^^,^^18^ This process has been described in cases involving the abdominal wall, pancreas, liver, and urachal remnant, explaining how a small linear foreign body can ultimately manifest as an apparent neoplasm distant from the site of initial perforation.^16–18^

Foreign body-related inflammatory processes have been described as deceptive tumor mimics in the literature. Goodman et al^19^ reported a gallstone-related phlegmon that closely resembled soft-tissue sarcoma on imaging, demonstrating how retained foreign material or calculi can generate mass-like appearances highly suggestive of malignancy.

Published reports of fish bone-related phlegmon demonstrate that this entity may masquerade as malignancy across a wide range of anatomical locations. Lesions have been initially interpreted as colorectal cancer, gastric submucosal tumors, pancreatic carcinoma, hepatic cholangiocarcinoma, and urachal carcinoma. Table 1 summarizes the published cases reporting tumor-like presentations secondary to fish bone perforation.^12^^,^^13^ This diversity of tumor-like presentations highlights the capacity of a small linear foreign body to produce radiological appearances that are indistinguishable from neoplasia.

**Table 1:** Tumor-like presentations of fish-bone perforation reported in the literature.

Location	Tumour-like presentation	Reference
Colon/abdominal wall	Fish-bone perforation mimicking colon cancer	Sibanda et al^7^
Cecum/abdominal wall	Cecal perforation causing an abdominal-wall abscess	Kuwahara et al^4^
Anterior abdominal wall	Anterior abdominal-wall abscess containing a fish bone	Nolasco Vaz et al^5^
Colon/abdominal wall	Colonic perforation with abdominal-wall abscess on imaging	Izniza et al^12^
Anterior abdominal wall	Abdominal-wall abscess mimicking a malignant tumour on CT, MRI, and PET CT	Kurose et al^9^
Colon	Sigmoid colon perforation by fish bone mimicking colorectal cancer	Hernández et al^13^
Stomach	Gastric pseudotumour caused by an embedded fish bone mimicking a gastric submucosal tumour	Kim et al^6^
Stomach	Fish-bone perforation of stomach mimicking a gastric submucosal tumour	Bajwa et al^14^
Stomach/pancreas	Fish-bone perforation of the stomach mimicking a locally advanced pancreatic carcinoma	Goh et al^15^
Stomach/pancreas	Pseudotumoural gastric lesion due to fish bone perforation	Al-Deeb et al^16^
Pancreas	Pancreatic abscess secondary to embedded fish bone presenting as a pancreatic mass	Wu et al 2022^17^
Liver	Inflammatory pseudotumour of the liver caused by a migrated fish bone, initially suspected to be cholangiocarcinoma	Liu et al^18^
Urachus/midline abdominal mass	Fish-bone perforation into a patent urachus mimicking urachal carcinoma	Alanazi et al^19^

### Abdominal wall mass differential and mimics

The differential diagnosis of an abdominal wall mass includes desmoid tumors, metastases, primary soft-tissue sarcoma, endometriosis, hernias, and inflammatory masses, many of which share overlapping imaging features.^2^ Chronic inflammatory processes such as phlegmon or abscess may manifest as mass-like enhancing lesions with surrounding edema, closely mimicking malignant features.^11^

Across reported cases of fish bone-related phlegmon, a consistent imaging pattern has been described. This typically comprises a localized mass-like lesion with a central low attenuation or fluid component, peripheral rim enhancement, surrounding inflammatory fat stranding, and a linear hyperdense foreign body measuring approximately 2 to 3 centimeters on CT (ie, the fishbone).^4–7^^,^^9^ The tumor-like appearance of these lesions contributes to diagnostic confusion and inappropriate oncologic referral.

### Imaging hallmarks of sarcoma and role of radiological modalities

CT features that raise concern for soft-tissue sarcoma include a rapidly growing heterogeneous soft-tissue mass, areas of necrosis or hemorrhage, irregular or lobulated margins, and variable enhancement patterns.^1^^,^^2^ On MRI, sarcomas typically demonstrate low to intermediate signal intensity on T1-weighted sequences, high signal intensity on T2-weighted sequences, heterogeneous internal architecture, necrotic or myxoid components, and variable contrast enhancement.^1^ Imaging features associated with malignancy include size greater than 5 cm, deep location, and progressive enlargement.^1^ Calcification or mineralization may be seen in certain soft-tissue sarcomas, particularly synovial sarcoma and extraskeletal osteosarcoma. However, malignant mineralization typically appears as punctate, amorphous, peripheral, or irregular foci, in contrast to the solitary thin curvilinear hyperdensity characteristic of a retained fish bone.^1^^,^^20^

Ultrasound is useful for assessing superficial abdominal wall lesions but is limited in detecting small or deeply located calcified foreign bodies.^2^ CT remains the most sensitive modality for identifying fish bones, as consistently demonstrated across reported cases.^4–7^^,^^9^ CT allows accurate delineation of extraluminal migration and inflammatory extension. Best practice includes thin slice acquisitions of 1 to 2 mm, review using bone window settings, and multiplanar reformats.

MRI is valuable for staging soft-tissue tumors and evaluating fascial planes.^1^^,^^2^ However, fish bones are often poorly visualized on MRI, and associated inflammatory lesions may appear solid, complex, and tumor-like, reinforcing a neoplastic differential.^6^^,^^9^ FDG PET CT lacks specificity, as inflammatory phlegmon may demonstrate intense fluorodeoxyglucose uptake, which may further confound interpretation.^9^

## Practical radiological lessons and diagnostic considerations

Although the clinical and laboratory findings in all cases were consistent with an inflammatory or infective process, the identification of a discrete soft-tissue mass on imaging generated sufficient diagnostic uncertainty to prompt referral to a regional Soft-Tissue Tumor unit for specialist assessment. These cases illustrate several practical diagnostic lessons highlighted by sarcoma specialists at a tertiary referral center.

First, all cases demonstrated inflammatory clinical features, including pain, local tenderness, raised inflammatory markers, and a waxing and waning course, with at least 1 patient showing partial response to antibiotic therapy. Such features are atypical for soft-tissue sarcoma and should raise suspicion for an inflammatory or infective process. The principal malignant exception would be a fistulating gastrointestinal stromal tumor, which remains rare. Furthermore, imaging characteristics such as surrounding fat stranding and inflammatory edema, the presence of a central fluid component with peripheral rim enhancement, and interval reduction in lesion size following antibiotic therapy further support an inflammatory or infective etiology.

Second, recognition of the so-called fish bone sign is pivotal. Identification of a single, thin, curvilinear hyperdense structure on CT is diagnostic of a retained foreign body. Malignant calcification does not present in this configuration and is never solitary and curvilinear. Once this feature is identified, diagnostic uncertainty is effectively resolved.

Finally, lesion location provides an important contextual clue. All cases were confined to the abdominal wall and demonstrated anatomical continuity with an adjacent bowel loop, consistent with transmural perforation and foreign body migration. While gastrointestinal stromal tumors (GISTs) originate from the bowel wall and may appear closely associated with bowel loops, primary soft-tissue sarcomas of the abdominal wall typically arise independently of the bowel. Therefore, a lesion demonstrating direct continuity with bowel should prompt consideration of alternative diagnoses. Colonic malignancies may fistulate through the abdominal wall but rarely do so at presentation. Careful assessment of lesion location and adjacent structures is therefore essential in recognizing this important sarcoma mimic.

## Conclusion

Fish-bone perforation of the gastrointestinal tract is an uncommon but important cause of abdominal wall phlegmon that may closely mimic soft-tissue sarcoma on imaging. Recognizing this entity and maintaining it within the differential for atypical abdominal wall masses can prevent unnecessary biopsy, oncologic referral, or major surgery. Radiologists play a central role in avoiding misdiagnosis by integrating multimodality imaging findings with clinical context and, where appropriate, MDT discussion.
